# Breaking Conventional Eating Habits: Perception and Acceptance of 3D-Printed Food among Taiwanese University Students

**DOI:** 10.3390/nu16081162

**Published:** 2024-04-13

**Authors:** Min-Yen Chang, Wei-Jiun Hsia, Han-Shen Chen

**Affiliations:** 1 Department of Accounting, Jiaxing University, Jiaxing 314001, China; mingyen0223@zjxu.edu.cn; 2 Department of Health Industry Technology Management, Chung Shan Medical University, Taichung City 40201, Taiwan; annie8260@gmail.com; 3 Department of Medical Management, Chung Shan Medical University Hospital, Taichung City 40201, Taiwan

**Keywords:** novelty food, 3D-printed food, food neophobia, sensory appeal, perceived health risk

## Abstract

Considering the prevalent strain on environmental resources imparted by existing food systems, prioritizing environmental sustainability is an imperative course of action. Subsequently, the shift towards sustainable production and consumption patterns engenders an escalating demand for environmentally conscious food systems. Thus, 3D-printed food technology surfaces are a promising solution noted for their efficacy in curtailing food waste, bolstering environmental sustainability, and imparting innovative strategies to the food supply chain. Herein, we amalgamate the theory of planned behavior (TPB) framework with several variables, namely ‘sensory appeal’, ‘food neophobia’, ‘perceived health risk’, and ‘environmental friendliness’ to probe the behavioral intentions of Taiwanese university students’ perceptions about 3D-printed food. Employing the snowball sampling method, 370 questionnaires were disseminated, out of which 319, constituting an effective retrieval rate of 86.2%, were deemed valid. Statistical analysis produced intriguing findings. Consumers’ inclination to purchase 3D-printed food is substantially determined by their attitudes, subjective norms, sensory appeal, food neophobia, perceived health risks, and environmental friendliness. Contrary to our initial hypothesis, perceived behavioral control did not exhibit a significant impact on consumers’ propensity to purchase 3D-printed food. Therefore, businesses should focus on magnifying the sensory appeal of 3D-printed food, coupled with precise nutritional labeling, to bolster consumer interest, enhance acceptance, and augment behavioral intentions. This study sheds light on the potential for the development of 3D-printed food in Taiwan, providing an indispensable reference for future endeavors in Taiwan’s 3D-printed food industry.

## 1. Introduction

The current era is witnessing a burgeoning global temperature, along with an augmented frequency of adverse climatic phenomena, jeopardizing the stability of agricultural food systems [1]. Food systems are inundated with environmental degradation and stress [2,3], raising concerns such as greenhouse gas emissions, biodiversity loss, land-use alterations, and chemical pollution [4]. These escalating concerns necessitate prioritizing environmental sustainability as an amplified global issue. Consequently, in 2015, the United Nations (UN) proposed sustainable development goals (SDGs), encompassing 17 primary objectives and 169 specific targets [5]. The first two goals, ‘No Poverty’ and ‘Zero Hunger’, closely align with the development and evolution of industries, such as food systems, agriculture, and food manufacturing [6].

According to statistics from the Taiwan Executive Yuan Ministry of Agriculture, the self-sufficiency rate for food in Taiwan was 30.7% in 2022 [7]. From an environmental standpoint, Taiwan’s food supply pivots heavily on imports, exacerbating the carbon footprint attributable to the food transportation process. Consequently, implementing sustainable production and consumption patterns to alleviate environmental strain has propelled policy trends [2], increasing the demand for sustainable food systems [4]. The fundamental challenge is establishing strategies that cater to contemporary demands, while preserving the requirements of future generations [8]. Such strategies may potentially involve innovative food varieties [9].

Novel foods are either derived from traditional food ingredients or produced using unconventional food processing methods, such as insect-based foods, lab-grown meat, and 3D-printed foods [10,11]. These foods are potential sustainable dietary options under the premise of attenuating environmental stress [9]. Insect-based foods present ample evidence in this regard, with their abundant protein content and advantageous position in reducing greenhouse gas emissions compared to animal and poultry meat [11,12]. Alternative meat varieties, such as lab-grown meat, offer effective strategies for alleviating environmental stress and improving animal welfare [13]. Nevertheless, 3D-printed food technology has emerged as a game-changer, minimizing food waste, while fortifying environmental sustainability within food storage, transportation, preparation, and reuse of food waste [14]. Hence, investigating 3D-printed food technology with an efficient food production process and potential contribution to achieving the 2030 sustainability goals forms the original intention of this research.

Leveraging the theory of planned behavior (TPB), this study further dissects the motivations and factors governing human behavior concerning 3D-printed foods [15]; this framework posits that behavioral intent leans on three cornerstones, as follows: attitudes, subjective norms, and perceived behavioral control [15]. The TPB has found widespread application in the environmental behavior research sector to gauge personal attitudes and intentions towards environmental conduct [16,17,18], waste reduction, energy conservation, sustainable nutrition behaviors, and acceptance and consumption intention of innovative foods [19,20]. Menozzi [21] employed the TPB to predict consumers’ willingness to consume flour products containing insects as ingredients. Zhang [22] surveyed the consumer acceptance of lab-grown meat using the same theory.

This study aimed to expand the understanding of consumer behavior towards 3D-printed food by applying the theory of planned behavior (TPB) to assess and predict behavioral intentions related to the purchase and acceptance of this innovative culinary technology. While prior research has illuminated consumer acceptance of novel foods, our focus is as follows:

1. Evaluating consumer willingness to try 3D-printed food, based on endorsements by experts and scholars.
2. Assessing the likelihood that consumers will recommend 3D-printed food to their personal networks after personal experience.
3. Investigating key variables driving consumer perception—food neophobia, sensory appeal, perceived health risks, and environmental considerations.

Our survey, tailored around these specific parameters, sought to capture the nuances of Taiwanese college students’ responses to 3D-printed food. We aimed to unravel the factors that might influence their readiness to incorporate such food into their diet, with an emphasis on uncovering any hesitations rooted in novelty aversion, health concerns, or environmental motivations.

The insights gleaned from this research will be instrumental in strategizing marketing approaches for 3D-printed food in Taiwan and in anticipating its market trajectory. By enhancing the understanding of consumer attitudes and potential acceptance, we anticipate valuable guidance for the 3D-printed food industry’s future development and innovation pathways.

## 2. Literature Review and Hypothesis Development

### 2.1. Theory of Planned Behavior

The TPB, an extension of Ajzen’s [23] theory of reasoned action, offers a theoretical schema asserting a correlation between behavioral intention and concrete enactment. The TPB underscores that positive attitudes and subjective norms, in tandem with a robust perception of controlling behavior, enhance an individual’s intent to perform the expected behavior [24]. Given sufficient control over a particular behavior and entrenched behavioral intent, individuals actualize their behavior when an opportunity arises [24]. Consequently, this study adopted the TPB as its foundation and proceeded with an empirical analysis, incorporating research variables such as consumers’ attitudes towards innovative food, perception of social pressure (emanating from professionals or family/peers), perceived behavioral control concerning the consumption of 3D-printed food, and intent to purchase the same.

#### 2.1.1. Attitude

Ajzen [15] treats attitude as a product of an individual’s appraisal of their behavioral beliefs and consequential evaluations [25], effectively indicating an individual’s positive or negative evaluation of a specific object. Positive attitudes towards a behavior typically result in a higher inclination to performance, whereas negative attitudes lead to a lower inclination. Research corroborates the idea that green attitudes significantly affect an individual’s sustainable purchasing intent [26]. Specifically, consumers who place a high premium on health often exhibit a positive attitude towards organic food, escalating their intent to purchase [27,28,29,30]. Drawing from these theories and the literature, this study formulates Hypothesis 1, as follows:

**H1:**  *Consumers’ positive attitudes towards 3D-printed food significantly and positively influence their behavioral intentions.*

#### 2.1.2. Subjective Norms

Subjective norms, as conceptualized by Ajzen [15], refer to the perceived social pressure individuals experience from influential individuals or groups during their decision-making processes. Onwezen et al. [31] highlighted subjective norms as pivotal determinants in predicting consumer intent, a finding validated across fields involving artificial burgers, fish, seaweed, bean, and insect burgers [32]. Jensen and Lieberoth [33] established that subjective norms play a crucial role in predicting the consumption behavior of breadworms; in their study on cricket consumption, Gumussoy and Rogers [34] asserted that subjective norms significantly influence the acceptance and consumption of unfavorable food. A positive correlation emerged between consumers’ attitudes and subjective norms in influencing their purchase intentions [35]. Following the literature and theoretical analysis, Hypothesis 2 is proposed as follows:

**H2:**  *Consumers’ subjective norms significantly and positively influence their purchase intentions for 3D-printed food.*

#### 2.1.3. Perceived Behavioral Control

According to Ajzen [23], perceived behavioral control demonstrates the ease or difficulty perceived by individuals in conducting a specific behavior. Influences on this perception are subjective and may include past experiences, anticipated obstacles, or personal objectives, as well as other resources such as self-capability, time, money, skills, opportunities, resources, and policies. Mancini et al. [36] utilized perceived behavioral control as a predominant predictor of intent-based factors, while other studies have confirmed its significant positive impact on individual behavioral intentions [37,38,39]. Pennanen et al. [40], in their research on the consumption of plant-based cheese, found that positive attitudes and perceived behavioral control enhance consumption willingness. Laksmawati et al. [41] observed, in their study on Taiwanese consumers, that information about the carbon footprint of product packaging can influence consumer behavior. For instance, pork has a lower carbon footprint than beef, leading to a dwindling popularity of beef and a simultaneous increase in that of pork. However, the question of whether consumers base their choices on personal dietary preferences and desires remains unanswered. Accordingly, Hypothesis 3 was formulated as follows:

**H3:**  *Perceived behavioral control significantly and positively influences consumers’ purchase intentions for 3D-printed food.*

### 2.2. Sensory Appeal (SA)

Emotional reactions significantly affect consumers’ food acceptance or rejection, with disgust typically resulting in outright refusal. Reactions are often derived from the sensory properties of the food items in question [34]. As a primary factor considered during food selection, sensory characteristics assume heightened significance in novel foods unfamiliar to consumers [42]. In such cases, consumers rely heavily on visual indicators to discern potential sensory traits [43].

Sensory appeal encompasses a broad spectrum that incorporates the visual, aromatic, and taste dimensions of foods [44]. Robust evidence attests to sensory characteristics as cardinal determinants in the purchase and consumption of organic and insect-based foods [45,46]. However, studies have argued that sensory appeal assessment transcends superficial considerations and involves a complex interplay of subjective consciousness, internal perceptions, and behaviors [47]. Consequently, Hypothesis 4 was as follows:

**H4:**  *Sensory appeal exerts a significant positive impact on purchasing intent towards 3D-printed foods.*

### 2.3. Food Neophobia (FN)

Food neophobia, defined as a consistent trait indicating an individual’s reluctance to consume unfamiliar or novel foods, is modulated by various factors including age, sex, income, educational level, family history, and the frequency and volume of food consumption [48,49,50], potentially impinging on healthy dietary patterns [51]. Notably, consumers exhibiting food neophobia often harbor negative predispositions towards novel food types, distinguishing them from those who are not resistant to novelty [52].

Such reluctance extends to the refusal to purchase or consume novel food varieties with unfamiliar raw materials, notably algae, plant-based meat replacements, and beans [34]. Consequently, it is essential to evaluate consumers’ propensity for food neophobia to create suitable food types and design strategies for 3D food printing [51]. Studies have affirmed the value of minimizing food neophobia in enhancing consumer acceptance of insect consumption [53].

Although several studies have reported a negative correlation between food neophobia and purchasing behavior, the critical role of sociocultural factors cannot be overlooked. Mancini et al. [54] revealed a higher acceptance of insect consumption in Northern Europe than in Central Europe or the Mediterranean regions, because the northern areas were pioneers in introducing edible insects to the market. Similarly, regions in northern and Central-West Brazil reflected more receptive attitudes towards insect consumption, due to Native American cultural influences, than their northeastern, southeastern, and southern counterparts [55]. Considering these insights, Hypothesis 5 was formulated.

**H5:**  *Consumer food neophobia significantly and negatively affects willingness to purchase 3D-printed food.*

### 2.4. Perceived Health Risk (PHR)

Data from the World Health Organization specify foodborne illnesses as the principal cause of mortality due to food consumption, claiming an estimated 33 million lives worldwide, due to unsafe food handling and consumption [56]. Recently, Taiwan has witnessed recurrent food safety incidents ranging from melamine milk powder scandals to adulterated oil episodes. Such events have heightened the scrutiny of Taiwanese consumers’ applications in food safety and associated health risks.

Compounded by the global nuances of the COVID-19 pandemic, there has been great concern regarding the possibility of indirect pathogen carrier transmission through food or contaminated food packaging [57]. Taken together, these factors augment the perception of health risks in food choices. Various studies have shown that perceived health risks are a determinant of the acceptance of novel plant-based products [31,58].

According to the theory of perceived risk, consumers sense potential risks during the decision-making process, because uncertainty can precipitate undesired repercussions [59]. Research on consumer perceptions of 3D-printed meat and insect-based products indicates that 3D-printed food is unnatural, potentially harmful, lacks freshness, tastes bad, or is deficient in nutritional value [14,60]. Additionally, increasing awareness of food health has escalated skepticism towards the edibility of insects, associating insect consumption with contracting infectious diseases and forming negative impressions [61]. Considering these insights, Hypothesis 6 is formulated as follows:

**H6:**  *Perceived health risks significantly and negatively affect consumers’ purchasing intentions for 3D-printed foods.*

### 2.5. Environmentally Friendly (EF)

Environmental psychology underscores individuals’ environmental identification or their nexus with nature as a pivotal driver that regulates environmental behavior in the context of environmental preservation [62]. The UN’s statement of the SDGs in 2015 kindled the heightened awareness of sustainability and underscored the importance of sustainable environmental conduct [63]. This surge in awareness has invariably fortified the human–environment relationship and paved the way for a host of EF behaviors, such as the adoption of reusable cups.

EF behavior encompasses actions that directly contribute to environmental betterment or mitigate adverse environmental impacts [64]. Notably, 3D-printed food, with its amalgamation of creativity, convenience, health, waste reduction, and enhanced environmental sustainability, represents an innovative step in food science [65]. These environmentally amicable food consumption behaviors include a reduction in animal product consumption, a decrease in food waste [66], and an increased prevalence of seasonal and local production [67,68].

Hartmann et al. [69] observed a salient correlation between consumer evaluations of a product’s environmental friendliness, health, and naturalness, suggesting an inherent relationship among these factors in consumer consciousness. Therefore, Hypothesis 7 is proposed, as follows:

**H7:**  *Environmentally friendly consumers have a significant positive impact on their behavioral intentions toward* *s 3D-printed food.*

## 3. Research Methodology

### 3.1. Research Framework

**Figure 1:**
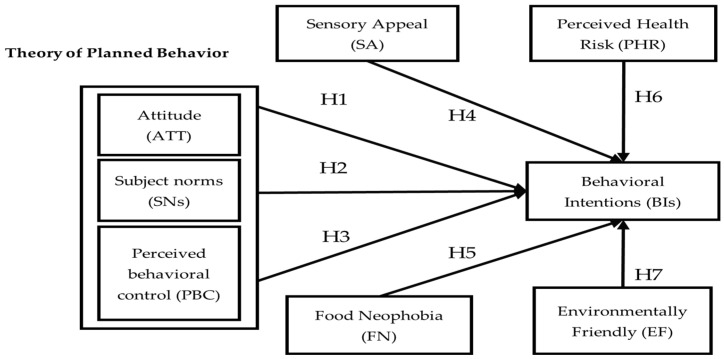
Research framework diagram.

Our research, based on the TPB theory, encompasses the following four variables: sensory appeal, food neophobia, perceived health risk, and environmental friendliness. It scrutinized Taiwanese university students’ perceptions of and attitudes towards 3D-printed food and predicted consumer purchasing behavior. The research framework is illustrated in Figure 1.

### 3.2. Survey Methodology

Despite its nascent stage in Taiwan, with minimal studies concerning 3D food printing [70,71] and no commercialized products in the market, we designed a method to familiarize respondents with 3D-printed foods. Before engaging in the questionnaire, respondents were shown an informative video on 3D-printed food, elucidating the incentive for developing novel food, and defining 3D-printed food within novel food [10,11]. The video further explored the nature, processes, and extant varieties of 3D-printed food.

The questionnaire, structured into nine sections, addressed attitudes based on Menozzi et al. [21], Sultan et al. [72], Tesikova et al. [73], and Govaerts and Olsen [74]; subjective norms based on Sultan et al. [72] and Chen [75]; perceived behavioral control following Sultan et al. [72] and Lee et al. [51]; sensory appeal derived from Imtiyaz et al. [76]; food neophobia following Pliner and Hobden [77], Siegrist et al. [50], Jaeger et al. [78], and Lee et al. [51]; perceived health risk based on Hwang and Choe [79] and Tesikova et al. [73]; environmental friendliness following Verain et al. [80]; and behavioral intentions derived from Lee et al. [51] and Tesikova [73] and demographic variables. Each variable was assessed using a seven-point Likert scale, allowing respondents to express their perspectives authentically.

To ensure the validity and reliability of the questionnaire and eliminate ambiguity, we conducted an expert review, inviting nine experts with over ten years of experience in education and the food industry. We addressed their suggestions, conducted a pre-test with 95 distributed questionnaires, and received 92 valid responses, indicating the robustness, construct validity, and reliability of the survey.

### 3.3. Sample and Data Collection

Owing to the convenience and pervasiveness of the internet, our research adopted online surveys via email, social media channels, personal contacts, and online communities. Despite the lower response rate than that of paper-based surveys, online distribution assists in augmenting the research reach, bolstering the completeness of responses and conserving resources.

For data analysis, we used structural equation modeling (SEM) to determine the optimal sample size using the ratio of the sample size to the number of items [81]. This study conducted surveys from November 2023 to December 2023, collected 370 forms, discarded 51 invalid responses, and compiled 319 valid responses.

### 3.4. Data Analysis Methods

This study utilized a quantitative research methodology, accumulating data through an online survey and performing data analysis using IBM SPSS Statistics version 28 and AMOS version 28 statistical software. The statistical analysis methods employed included descriptive statistics, reliability, validity analysis, maximum likelihood estimation of structural equations, analysis of the proposed hypotheses, overall model fitness, and assessment of the causal relationships of the hypothetical model.

## 4. Analysis and Results

### 4.1. Measurement Model: Reliability and Validity

Table 1 presents the meticulous analysis of the validity of each variable. If the factor loading of each construct exceeds 0.5, the variable has convergent validity [82]. The average variance extracted (AVE) serves as an indicator of convergent reliability. Internal consistency reliability was evaluated using Cronbach’s α, which denotes internal consistency if exceeding 0.70 [83]. Composite reliability estimates the internal consistency of a combination, with a minimum acceptable value of 0.70 [83]. Here, every factor load exceeded 0.5 and the AVE was 0.689–0.861, surpassing the minimum requirement of 0.5. Cronbach’s α values were 0.819–0.941 and composite reliability values were 0.773–0.926, exceeding the 0.7 threshold, suggesting high internal consistency among and composite internal consistency within the variables.

Discriminant validity was evaluated according to the guidelines of Hair et al. [83] to ascertain whether the measurement items were effectively distinguished between varied constructs. Discriminant validity ensures that the correlation coefficient between different variables is less than the square root of their respective AVE, suggesting diverse validity levels among the research variables. Table 2 illustrates the comparison of the mean, standard deviation, correlation coefficients, and square root of the AVE of all variables, with the square root of each variable’s AVE exceeding its inter-variable correlation coefficients, conforming to Hair et al.’s [83] standard.

### 4.2. Model Fit Verification

A confirmatory factor analysis was performed using AMOS 28.0. The study found χ^2^/df (2.314) and RMSEA (0.064) values below the recommended thresholds of 3 and 0.08, respectively [84]; AGFI (0.837) values exceeded the 0.8 standard of 0.05 [85]; NFI (0.907), CFI (0.944), and IFI (0.945) values surpassed 0.9 [86]; however, RMR (0.96) fell short of the 0.05 requirement [84].

### 4.3. Overall Model Path Analysis

**Figure 2:**
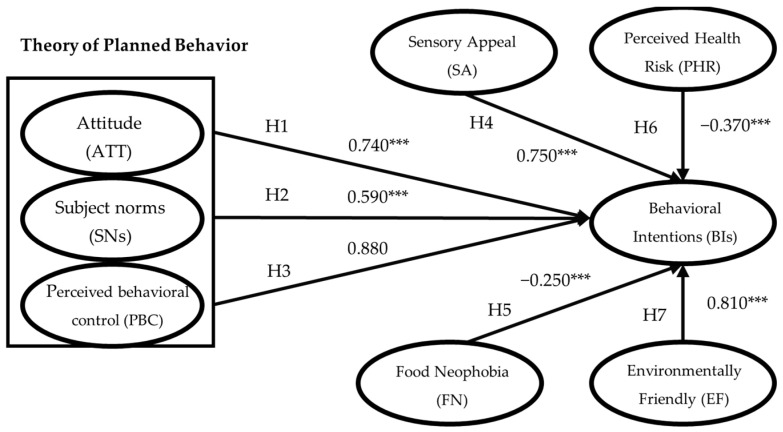
Structural equation modeling diagram. Note: *** *p* < 0.001.

Inter-variable relationships were examined using a structural equation model (Figure 2). H1 outlined a significant positive correlation between consumer attitudes and behavioral intentions toward 3D food (β = 0.740, *p* < 0.001). H2 postulates that consumers’ subjective norms significantly influence 3D-printed food behavioral intentions (β = 0.590, *p* < 0.001). H3 suggested a significant positive influence of perceived behavioral control on behavioral intentions (β = 0.880, *p* > 0.01). H4 argues that sensory appeal significantly affects behavioral intentions (β = 0.750, *p* < 0.001). Finally, H5 indicated that food neophobia negatively affects behavioral intentions for 3D-printed food (β = 0.590, *p* < 0.001).

Thus, H1, H2, and H5 are substantiated and significant; H4 is substantiated but insignificant; and H3 is not substantiated. Table 3 summarizes the pathway and hypothesis-testing results.

## 5. Discussion

This study meaningfully contributes to the discourse on consumer behavior, particularly highlighting the emergent inclination towards sustainable purchasing practices, through the lens of 3D-printed food technology. Echoing Shehawy [26], we affirm that pro-environmental attitudes significantly influence sustainable behavioral intentions. This is also reflected in the seminal work of Ajzen’s theory of planned behavior [23], which posits that behavioral intentions are driven by attitudes, subjective norms, and perceived behavioral control. Our research contributes a novel perspective by illustrating that, in the context of innovative food solutions, such as 3D-printed food, environmental consciousness can play a critically transformative role. Nonetheless, this challenges part of Ajzen’s model by revealing that perceived behavioral control may not be a significant factor in the adoption of this technology. This deviation from established behavioral norms requires further scrutiny.

Alzubaidi et al. [87] revealed that environmental concerns form a precursor to eco-friendly behaviors. This underscores the need to cultivate such attitudes, which, as our study shows, could foster favorable consumer perceptions of pioneering food technologies. Additionally, Onwezen et al. [31] emphasized the influence of authoritative endorsements on consumer decisions, suggesting a practical stream for stakeholders—leveraging expert opinions can mold a receptive market for technologies such as 3D-printed food, where subjective norms heavily sway consumer purchase intentions.

Analogous to the findings of Nie et al. [88], our research acknowledges the complexity of consumer decision-making, noting how multiple concerns, from health to ethics, intersect to shape consumer behavior. This confirms a synergy between consumer attitudes towards health, the environment, and ethical considerations and their purchasing behavior, lending credence to the integrative role these factors play in influencing consumer choices.

In addition to theoretical complexity, the literature indicates that high product quality perceptions encompassing attributes such as naturalness and purity heighten consumers’ propensity to engage in green purchases [89,90]. However, when consumers’ self-efficacy regarding behavior execution is low, as identified by Hidayat et al. [91], the robustness of perceived behavioral control in predicting actual behavior is attenuated. This nuanced relationship necessitates an extended investigation into how self-efficacy may enhance or deter the purchase of 3D-printed food products.

Weaving in Xu et al. [92], consumer awareness of multiple dimensions such as health and food safety uniquely shapes preferences. This highlights the possibility of insufficient consumer knowledge about 3D-printed food technology, which may lead to an inability to thoroughly evaluate product quality, signaling a gap ripe for educational intervention.

Our study acknowledges that the nascent stage of 3D-printed food technology may lead to hesitation among consumers, chiefly because of a lack of familiarity and regulatory oversight, as emphasized by Hojnik et al. [93]. Findings from a specific cohort of Taiwanese university students underline this sentiment, highlighting the broader theme of systemic distrust that encompasses new technologies.

Our discussions have illustrated how sensory compatibility with conventional food can attenuate barriers to acceptance, echoing the viscerally driven consumer choices documented by Magnusson et al. [45]. We concur with Ruiz-Capillas et al. [94] in that the sensory appeal significantly influences consumers’ purchasing intent, reinforcing the importance of sensory compatibility with existing food norms. Juxtaposed with the challenge of neophobia highlighted by Jang and Kim [52], scaling nontraditional food technologies in the marketplace is an appreciable hurdle.

Guzek et al. [95] suggested that the cultural domain is substantial in food-related attitudes, thus accentuating the necessity for an inclusive and culturally sensitive approach when considering the global expansion of 3D-printed food products. These nuances must be factored into future deployment strategies to ensure broader acceptance of the technology.

Aligning with Shin and Kang [59], our investigation uncovers consumer perceptions of health risks, which might overshadow enthusiasm toward novel food technologies. Hartmann et al. [69] and Krettenauer et al. [62] demonstrated a positive correlation between environmental priorities and preference for sustainable food alternatives, marking a fertile territory for promoting 3D-printed food.

According to Pšurný et al. [96], heightened environmental sustainability awareness is a crucial driver of consumer purchasing behavior, which aligns with the dynamics of subjective norms and the endorsement effect observed in our study.

We ensure that our sample selection is robust and appropriately corroborated by Hair et al. [97] for suitable inferential analysis and recognize the unique implications stemming from Taiwanese students’ attitudes towards 3D-printed food technology. Future research should endeavor to bridge the gaps presented here, aiming to garner a global perspective on consumer receptivity to 3D-printed food technologies through cross-cultural and demographic comparisons.

In summary, this study sheds light on the multifaceted nature of consumer engagement with 3D-printed food and underlines the importance of continuing comprehensive and culturally sensitive research in this promising food technology sector.

## 6. Conclusions and Recommendations

To understand the factors influencing consumers’ intent to purchase 3D-printed food, this study effectively amalgamated sensory attractiveness, food neophobia, perceived health risks, and environmental friendliness concepts into a robust TPB framework. The interpretive nuances of consumer behavior revealed several important findings. A correlation exists among the critical revelations between consumers’ knowledge of environmental sustainability and their willingness to purchase 3D-printed food. Significantly, their commitment to environmental sustainability and the influence of sustainability indices promulgated by relevant organizations showed a substantial effect on their purchase decisions.

These results strongly advocate an enhanced focus on consumers’ societal and psychological contexts when developing strategies to stimulate sustainable consumption behavior. Ecological considerations are increasingly pivotal in shaping consumer choices, accentuating that a deeper understanding of these factors could greatly inform the market for environmentally innovative products such as 3D-printed food.

Given the potential of 3D-printed food materials within the Taiwanese food industry, the aesthetic and olfactory attributes of food must align with local culinary traditions and consumer preferences. To mitigate the potential negative impact of food neophobia, it is recommended that product labeling transparently elucidates all components and nutritional value, reducing the incidence of purchase refusal due to unfamiliarity or lack of product knowledge.

Whilst it is challenging, as it addresses the global urgency of the environmental sustainability agenda, consumer readiness to embrace sustainable development manifests itself as a beacon of hope. Remarkably, not all EF alternatives such as plant-based meat and milk are universally accepted. Considering the findings of this study, future research should extrapolate to the multifaceted aspects of consumer attitudes towards 3D-printed food, with analytical lenses focused on different age groups, religious cultural backgrounds (e.g., Christians), or occupational backgrounds. Additionally, future studies could benefit from incorporating supplementary variables, such as asymmetry of information and perception of health values, to gauge their influence on consumers’ procurement intentions. Such expansions would enhance the comprehensiveness of the research framework and offer a more holistic view of the dynamics involved in consumers’ intentions to purchase 3D-printed food.

## Figures and Tables

**Table 1:** Results related to factor loading, reliability, and validity.

Variables/Items	Standardized Factor Loading	AVE	CR	Cronbach’s α
Attitude (ATT)		0.689	0.917	0.885
1. I believe that 3D-printed food is edible.	0.721 ***			
2. I think 3D-printed foods are healthy.	0.863 ***			
3. I believe that the ingredients in 3D-printed food are natural.	0.856 ***			
4. I think 3D-printed food is harmless to the environment.	0.834 ***			
5. I believe that 3D-printed food is sustainable.	0.866 ***			
Subject norms (SNs)		0.680	0.864	0.762
6. Opinions of professionals such as nutritionists would affect my willingness to purchase 3D-printed food.	0.829 ***			
7. Opinions from individuals or peers would influence my desire to buy 3D-printed food.	0.847 ***			
8. Calls from environmental groups would affect my willingness to buy 3D-printed food.	0.797 ***			
Perceived behavioral control (PBC)		0.736	0.893	0.812
9. I can decide whether to choose 3D-printed food.	0.792 ***			
10. I am willing to pay more to purchase environmentally friendly 3D-printed food.	0.906 ***			
11. Eating 3D-printed food is a pleasure, if I have willingness.	0.871 ***			
Sensory Appeal (SA)		0.861	0.949	0.919
12. I would be willing to try it when the appearance of 3D-printed food is similar to that of familiar food.	0.913 ***			
13. I would be willing to try it when the smell of 3D-printed food is similar to that of familiar food.	0.950 ***			
14. I would be willing to try it when the taste of 3D-printed food is similar to that of a familiar food.	0.921 ***			
Food Neophobia (FN)		0.709	0.924	0.897
15. I do not trust novel foods.	0.861 ***			
16. I do not try novel foods.	0.837 ***			
17. The weird look of 3D-printed food makes me not eat it.	0.860 ***			
18. I have doubts about the hygiene and safety of 3D-printed food.	0.838 ***			
19. The ingredients of 3D-printed food make me suspicious.	0.813 ***			
Perceived Health Risk (PHR)		0.795	0.939	0.913
20. I worry that 3D-printed food is harmful.	0.898 ***			
21. I worry that 3D-printed food is unhealthy.	0.911 ***			
22. I believe that there is a risk of chemical contamination (e.g., heavy metals and pesticides) in 3D-printed food.	0.883 ***			
23. I believe that there is a risk of microbiological contamination (for example, E. coli and botulinum) in 3D-printed food.	0.873 ***			
Environmentally Friendly (EF)		0.744	0.921	0.884
24. I think 3D-printed food can reduce food waste.	0.803 ***			
25. I believe that 3D-printed food is a seasonal product.	0.884 ***			
26. I believe that 3D-printed food is a local product.	0.894 ***			
27. I think 3D-printed food is environmentally friendly.	0.866 ***			
Behavioral Intentions (BIs)		0.819	0.948	0.926
28. I have full confidence in 3D-printed food.	0.895 ***			
29. It is very likely that I will purchase 3D-printed food.	0.918 ***			
30. I would recommend that others buy 3D-printed food.	0.919 ***			
31. I believe I can buy 3D-printed food for reasons of its health and nutritional value.	0.887 ***			

Note 1: CR: composite reliability; AVE: average variance extracted. Note 2: *** *p* < 0.001.

**Table 2:** Discriminant validity rest.

	Mean	Standard Deviation	1.	2.	3.	4.	5.	6.	7.	8.
1. ATT	4.213	1.137	0.958	0.494 **	0.589 **	0.677 **	−0.336 **	−0.321 **	0.680 **	0.726 **
2. SNs	4.974	1.030	0.494 **	0.930	0.515 **	0.563 **	−0.81	−0.05	0.520 **	0.507 **
3. PBC	4.726	1.024	0.589 **	0.515 **	0.945	0.647 **	−0.202 **	−0.147 **	0.634 **	0.712 **
4. SA	4.863	1.212	0.677 **	0.563 **	0.647 **	0.928	−0.366 **	−0.229 **	0.658 **	0.704 **
5. FN	4.431	1.170	−0.336 **	−0.81	−0.202 **	−0.366 **	0.842	0.742 **	−0.270 **	−0.345 **
6. PHR	4.897	1.120	−0.321 **	−0.05	−0.147 **	−0.229 **	0.742 **	0.892	−0.269 **	−0.339 **
7. EF	4.491	1.163	0.680 **	0.520 **	0.634 **	0.658 **	−0.270 **	−0.269 **	0.863	0.768 **
8. BI	4.094	1.314	0.726 **	0.507 **	0.712 **	0.704 **	−0.345 **	−0.339 **	0.768 **	0.905

Note 1: The value in bold font is the square root of the AVE; the non-diagonal numbers represent the correlation coefficients of each dimension. Note 2: ** *p* < 0.01.

**Table 3:** Results of the path analysis and confirmation of hypotheses.

Hypothesized Paths	Unstandardized Coefficient	S.E.	C.R.	*p*	Standardized Coefficients	β	Verification Results
H1: ATT → BIs	294.770	38.436	7.669	<0.001	0.740 ***	0.207	Supported
H2: SNs → BIs	252.619	35.200	7.177	<0.001	0.590 ***	0.017	Supported
H3: PBC → BIs	90.783	26.865	3.379	<0.001	0.880	0.267	Unsupported
H4: SA → BIs	389.350	42.014	9.267	<0.001	0.750 ***	0.145	Supported
H5: FN → BIs	−140.406	35.785	−3.924	<0.001	−0.250 ***	0.009	Supported
H6: PHR → BIs	−191.909	34.318	−5.592	<0.001	−0.370 ***	−0.120	Supported
H7: EF → BIs	413.697	45.364	9.120	<0.001	0.810 ***	0.324	Supported

Note 1: ATT = attitude, SNs = subjective norms, PBC = perceived behavioral control, SA = sensory appeal, FN = food neophobia, PHR = perceived health risk, EF = environmentally friendly, BI = behavioral intentions. Note 2: *** *p* < 0.001.

## Data Availability

The data supporting the findings of this study are not publicly available due to the dataset includes personal data from participants who consented under the condition of confidentiality and non-disclosure, we are obliged to uphold the privacy rights. However, the data are available from the corresponding author, H.-S.C., upon reasonable request and with respect to the aforementioned constraints.
